# The N3C governance ecosystem: A model socio-technical partnership for the future of collaborative analytics at scale

**DOI:** 10.1017/cts.2023.681

**Published:** 2023-11-14

**Authors:** Christine Suver, Jeremy Harper, Johanna Loomba, Mary Saltz, Julian Solway, Alfred Jerrod Anzalone, Kellie Walters, Emily Pfaff, Anita Walden, Julie McMurry, Christopher G. Chute, Melissa Haendel

**Affiliations:** 1 Research Governance & Ethics, Sage Bionetworks, Seattle, WA, USA; 2 Owl Health Works LLC, Indianapolis, IN, USA.; 3 Integrated Translational Health Research Institute of Virginia (iTHRIV), University of Virginia, Charlottesville, VA, USA; 4 Department of Biomedical Informatics, Stony Brook University, New York, NY, USA; 5 Institute for Translational Medicine, University of Chicago, Chicago, IL, USA; 6 Department of Neurological Sciences, College of Medicine, University of Nebraska Medical Center, Omaha, NE, USA; 7 University of North Carolina, Chapel Hill, NC, USA; 8 Center for Health AI, University of Colorado Anschutz Medical Campus, Aurora, CO, USA; 9 Schools of Medicine, Public Health, and Nursing, Johns Hopkins University, Baltimore, MD, USA

**Keywords:** Biorepository, data governance, ethics, COVID-19, CTSA, decision-making, regulatory support

## Abstract

The National COVID Cohort Collaborative (N3C) is a public–private–government partnership established during the Coronavirus pandemic to create a centralized data resource called the “N3C data enclave.” This resource contains individual-level health data from participating healthcare sites nationwide to support rapid collaborative analytics. N3C has enabled analytics within a cloud-based enclave of data from electronic health records from over 17 million people (with and without COVID-19) in the USA. To achieve this goal of a shared data resource, N3C implemented a shared governance strategy involving stakeholders in decision-making. The approach leveraged best practices in data stewardship and team science to rapidly enable COVID-19-related research at scale while respecting the privacy of data subjects and participating institutions. N3C balanced equitable access to data, team-based scientific productivity, and individual professional recognition – a key incentive for academic researchers. This governance approach makes N3C research sustainable and effective beyond the initial days of the pandemic. N3C demonstrated that shared governance can overcome traditional barriers to data sharing without compromising data security and trust. The governance innovations described herein are a helpful framework for other privacy-preserving data infrastructure programs and provide a working model for effective team science beyond COVID-19.

## Introduction

Since the dawn of science, much has been gained by investigations across knowledge boundaries and the promotion of team science [1,2]. Across the world, public–private partnerships and cross-sector collaborations are sought to address complex societal problems that no single organization can resolve alone. However, engaging in collaborative research requires enhanced sharing of data and insights. Indeed, sharing health data in support of translational research can increase collaboration, discovery, scientific accountability, transparency, and reproducibility, reducing costly redundancy and ultimately improving patient outcomes [3]. However, access to critical datasets is often limited due to logistical and economic concerns (related to the dataset size and needs for data curation, transfer, and harmonization), institutional culture and protocols (concerns over use/misuse of the data and loss of control over the data), insufficient technical proficiency of aspiring users, privacy concerns, and complexities of legal and regulatory obligations. Diverse approaches exploring a combination of technology and governance have been proposed but have yet to be nationally scaled for individual-level sensitive health data. The COVID-19 pandemic provided a powerful incentive to overcome the technical, operational, and cultural barriers to data sharing. The National COVID Cohort Collaborative (N3C) was born from the imperative to share national COVID-19-related data from electronic health records (EHR) quickly and efficiently [4]. The mission of N3C is “… to help save lives by enabling collaboration among clinicians, researchers, and data scientists to identify treatments and specialized care needs and thereby reduce the immediate and long-term impacts of COVID-19.”

N3C is a unique public–private–government partnership comprised of federal sponsors and funders (e.g., the National Center for Advancing Translational Sciences (NCATS); the Clinical and Translational Science Awards (CTSA) Program hubs; the National Center for Data to Health (CD2H); the Institutional Development Award Networks for Clinical and Translational Research (IDeA-CTR)); several data contributing institutions (e.g., state or regional Health Information Exchanges, hospitals/health systems, academic medicine, and clinical research networks), technology and commercial partners such as Palantir Technologies, Amazon Web Services, MDClone, and a large self-organizing community of researchers.

Building this shared data resource involved collectively deciding how the resource would be produced, operated, used, and sustained. It was not solely an impressive technical feat but also required overcoming policy and cultural barriers. Stakeholders formed focus groups to address challenges and develop innovative solutions. A Partnership and Governance Workstream was tasked with developing community-guiding principles, rules, policies, procedures, and oversight mechanisms for contributing to and using the data responsibly. The workstream focuses on 1) engaging contributing institutions for safe data transfer, 2) selecting data harmonization/curation methods, 3) obtaining data access/use and analysis approvals, 4) ensuring the appropriateness of results output for dissemination, and 5) ensuring fair authorship and attribution.

### Factors integral to governance

For all N3C-intended activities, the tenets of the Common Rule, the HIPAA privacy rule, and the FAIR data management and stewardship principles that data be Findable, Accessible, Interoperable, and Reusable needed to be upheld [5–7]. This workstream implemented a generalizable governance framework with terms and conditions supported by technology and oversight mechanisms. Data protection standards are adopted by default, contributing sites are not identified, and access is provisioned per project and available only for COVID-19-related research. Data cannot be extracted or downloaded. Contributing organizations and patients/subjects can expect the most conservative interpretation to be used.

In just 3 months, N3C progressed from conception to data ingestion, harmonization, and sharing. Since its inception, the partnership has grown to include 75 organizations contributing data from over 230 sites and over 4600 researchers. N3C has become the largest national public COVID-19 EHR data resource. As of September 2023, the N3C enclave included data from more than 20 million people, including over 8 million with a COVID-19 diagnosis. This unprecedented resource has enabled over 500 research projects. By quickly allowing access to answer questions (many of worldwide relevance), N3C has generated generalizable knowledge of COVID-19 shared through numerous publications and presentations. A similar repository with the necessary governance and controls to enable thousands of researchers with hundreds of vetted and IRB-approved protocols has never been accomplished in such a short time, if ever. N3C received the 2022 DataWorks! Grand Prize for Innovations in Data Sharing and Reuse [8].

None of this work was created in a vacuum. N3C leveraged the CTSA network, the IDeA Clinical & Translational Research Network (IDeA-CTR) Award program, Research Consortia (i.e., PCORnet, Observational Health Data Sciences and Informatics (OHDSI), Accrual to Clinical Trials (ACT) network, and the commercial partner TriNetX [9–12]), and efforts established before the pandemic’s onset (e.g., the OCHIN network of community health centers [13]). The resulting governance was inspired by Ostrom’s Principles on governing the Commons [14], the eMERGE network [15], the Accelerating Medicines Partnership® Program for Alzheimer’s Disease (AMP® AD) [16], and other emerging models of data governance [17,18]. The target data model is based on the open-science, open-software OHDSI model and the Observational Medical Outcomes Partnership (OMOP) methodology [19].

There were numerous decision points along the pathway to N3C. In this article, we describe the design and implementation of the N3C governance approach in practice. We also highlight lessons learned that may benefit other collaborative research efforts beyond pandemic emergency response.

### Elements of N3C Governance

**Figure 1.**
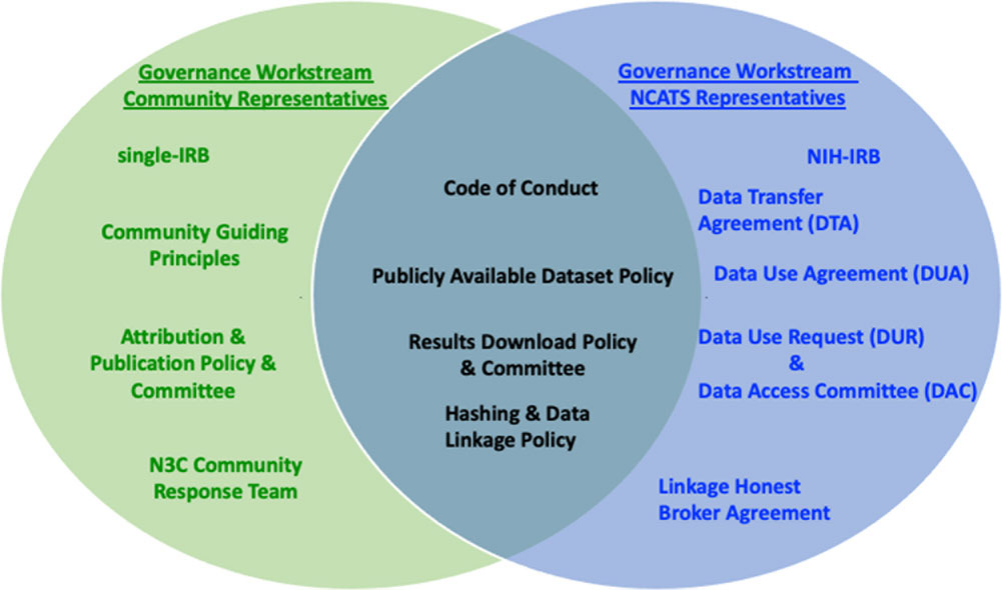
Equilibrium in governance. Network of shared governance initiatives with sign-off responsibility represented. NCATS and members of the Governance Workstream established the terms, behavioral expectations, and accountability mechanisms to enable N3C.

- **A decentralized form of rule-making and monitoring powered by broad community participation and reciprocity.** The **Partnership and Governance Workstream** established a governance model where NCATS engaged with the research community for all decision-making, even delegating final sign-off responsibilities to the Governance Workstream in some arenas (Fig. 1). Membership to the Workstream was open to representatives from every participating institution, with leadership provided by two Workstream Co-chairs and the N3C Co-Principal investigators (two academics and one at NCATS). Members developed the foundational behavioral and ethical expectations and norms that guide N3C [4], including the community principles, a diversity statement, and a conflict resolution process overseen by a Community Response Team. Terms for data contribution/transfer and data use to be executed between NCATS and participating institutions, and accountability mechanisms were established iteratively after consultations with the NIH Office of the General Counsel to respond to regulatory requirements and government practices while also addressing the needs of contributing institutions, researchers, data subjects, and communities. Workstream meetings were open to anyone interested in participating or monitoring activities. In the first year, 35–45 persons attended the weekly meetings, including representatives from participating sites specializing in technical, legal, and regulatory matters and data scientists and ethicists. Meetings were productive despite the large number of participants, likely reflecting the shared interests of stakeholders. Policies and procedures were posted on Zenodo for transparency and public comments [20].
- **A standardized data management process to generate a harmonized dataset devoid of readily identifying information protects the anonymity of data subjects and contributing institutions**. A unified and harmonized dataset was needed to provide usable data to researchers rapidly. The **Phenotype and Data Acquisition and the Data Ingestion and Harmonization Workstreams** were formed to determine how to select, ingest, and harmonize the wide variety of EHR datasets and conduct quality control. These workstreams chose OMOP 5.3.1 as a common data model for interoperability and established an orderly and standardized workflow to process the EHR data [21].

The established workflows left several critical technical and governance decisions in the hands of the contributing institutions. Data were accepted in one of four data models to maximize participation. Sites could perform modest random date shifting on each patient record before sending the data. Data enhancements (such as viral variant data and supplementary oxygen device data) were requested but not required. Contributing sites could also elect to provide hashed patient identifiers to a third-party honest broker, enabling Patient Privacy Preserving Record Linkage (PPRL) [22] to additional data such as mortality status and data from the Centers for Medicare & Medicaid Services (CMS). Source data model, date shifting range, and date of last extraction are provided to researchers in the enclave, but contributing sites are assigned an anonymous identifier. Geo-coded associations and temporal tracking were only possible with an approved IRB protocol justifying access to the limited dataset version. Although this flexibility required researchers relying on certain optional data enhancements to subset their analyses, the flexibility ensured maximum participation of contributing sites while respecting their local governance decisions.

- **Behavioral norms that reinforce research protections and accountability.** To access the enclave, investigators must follow several steps. Firstly, an official from their institution with signing authority must execute a Data Use Agreement (DUA) with NCATS. Secondly, data requesters must register with N3C, agree to a user Code of Conduct (CoC) [23], complete required ethical human subject and NIH information security training, and submit a Data Use Request (DUR) that describes how they intend to use the data and what level of data they wish to access. The CoC delineates the fundamental actions and prohibitions involving the use of N3C data and reflects the terms and conditions outlined in the DUA, including not attempting to identify contributing institutions, communities, or populations, not making assumptions about tribal affiliation, and abiding by the Community Guiding Principles and the Attribution and Publication Principles [24]. NCATS operates a Data Access Committee (DAC) charged with approving DURs. The DAC uses objective criteria to assess DUR, thereby promoting fair, equitable, and unbiased access to the resource.
- **A secure data enclave infrastructure that supports access to the data for analysis with strong security measures to protect the integrity of the data and prevent unauthorized data download.** N3C data are kept in a secure data enclave, hosted on an NIH-sponsored GovCloud instance that is FedRAMP [25] and FISMA [26] moderate level certified for compliance with high levels of physical and data security standards. Access to the data enclave requires (A) account creation and confirmation of investigator credentials by their sponsoring institution and (B) approval of a DUR by the federal DAC. Once in the enclave, the data are protected from disclosure by a Certificate of Confidentiality [27]. Recipients are bound by the terms of the Certificate and cannot redisclose the data except as permitted by the terms of the Certificate, including to Provider. Any copy of the data is still protected by the Certificate.

The data are compartmentalized into three levels of data access based on the risk of re-identification of data subjects:

1. Level one is for anonymous aggregate data and synthetic data where no actual data are available.
2. Level two is for HIPAA safe harbor de-identified data where dates are shifted and zip codes are anonymized as three-digit zip codes.
3. Level three is for HIPAA-limited datasets, including service dates and full zip codes.

**Figure 2.**
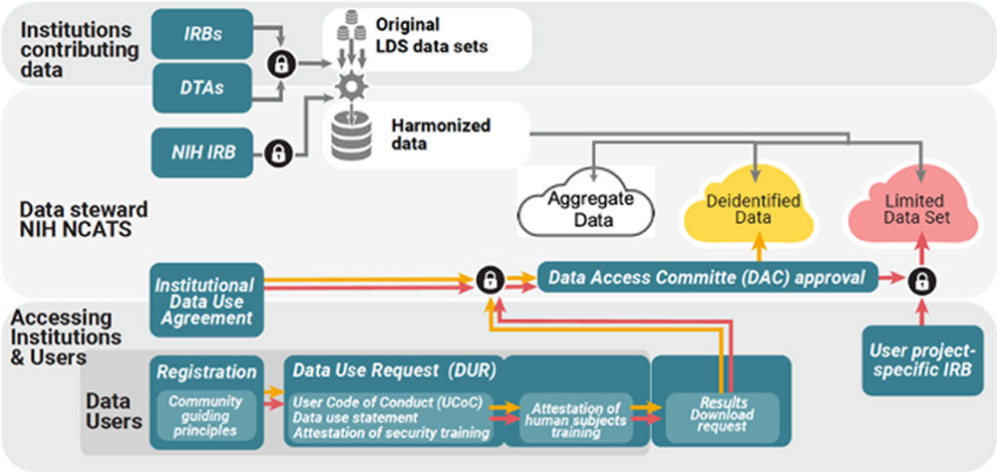
Steps for data contribution and use. Participating in N3C necessitates both institutional-level agreement(s) and user commitment. Institutions contributing data to N3C must obtain IRB approval and execute a Data Transfer Agreement with NCATS. Investigators wishing to access the enclave must ensure that their institution has executed a Data Use Agreement with NCATS. Investigators must agree to the N3C Community Guiding Principles and Code of Conduct, complete mandatory security and ethics training, and submit a Data Use Request (DUR) describing their project and the data level they wish to access. IRB approval is required to access HIPAA-limited datasets. An NCATS-administered Data Access Committee evaluates DURs before granting access to the level of data needed to accomplish the DUR. A Result Download Committee verifies that publications or presentations derived from N3C data do not contain patient or site-identifying information.

Investigators are granted access to the specific level of data needed for their approved analyses (Fig. 2). Access to the enclave requires multifactor authentication and activities are recorded and auditable. Researchers can conduct approved research within a dedicated analysis space; only analysis results, not individual data, are downloadable.

- **Domain teams to optimize analysis and cross-pollinate expertise across institutions.** N3C was designed to answer questions from various domains rather than a central clinical one to understand the disease. To avoid duplication of efforts and encourage team science, self-organized public “**Domain Teams**” were created, allowing researchers with similar interests to communicate and work together. Each team was encouraged to include diverse skill sets and expertise in informatics, data analysis, and clinical practices to ensure meaningful research questions. Over 30 volunteer-led domain teams have been established, which meet regularly to discuss goals and project design, review shared clinical concept sets, and present draft manuscripts for refinement. Meetings are open to attendees authorized to access data at the level being used or discussed during the meeting. Participating in a domain team helps new researchers get oriented to N3C and build from prior work before analyzing data in the enclave.
- **An authorship attribution and publication process that recognizes the impact of participants in team science and reinforces data privacy.** N3C aims to improve discoverability and accelerate access to research findings and analysis code. A community-led **Attribution and Publication Committee** was created to ensure that authors using N3C data uphold the N3C community guidelines and principles. While members of the Attribution and Publication Committee have broad-ranging expertise allowing them to provide feedback to the author for their consideration, the committee was not intended for scientific peer review. They review after download approval has been provided by the data stewards to ensure the following. The committee verifies adherence to the N3C policies and the approved DUR, promotes equitable and fair representation in authorship, and identifies analysis overlap. The committee also created a process for researchers to declare their contributions to manuscripts and established a consortial authorship for those authors who met the ICMJE guidelines [28].

Because the enclave disables data download, retrieving analytic results as parameters, tables, or figures requires special handling. A **Result Download Committee** was tasked with determining what aggregate data can be published and ensuring that no identifying information, and no results of fewer than 20 participants, are included in publications or presentations, without special authorization from NCATS. This committee is the N3C solution to the UK Five Safes Framework for safe outputs [29].

## Discussion

The N3C governance approach enabled the creation of an effective interdisciplinary partnership with checks and balances and technical means to address scientific questions.

Below we list several steps that were deemed critical for the successful replication of a central data repository from independent medical centers. It will likely be a new generation that faces another pandemic at the same scale and documenting these requirements will help guide future successful efforts to bring together a level of team science a pandemic justifies.

**1. Step to Success: Equilibrium in governance is central to N3C success but is hard to achieve**

To be successful, the Governance Workstream needed to nurture the tripartite public–private–government partnership and involve stakeholders in decision-making. The first challenge to establishing this governance approach was to recognize and balance each party’s motivation, roles, and responsibilities, agree on the scope of community versus federal authority, and set the groundwork for possible future expansion of the governance framework beyond N3C.

- As data steward, NCATS had fiduciary responsibility for the data enclave and adjudicating access.
- The researcher community wanted to define ethical values for N3C, establish collaboration rules, and ensure the scientific outputs’ quality.
- Data scientists developing methods and generating data ingest workflows brought scientific expertise beyond data coordination that needed to be recognized.
- Data-contributing institutions needed to protect their rights, interests, and contractual obligations.
- Researchers needed to promptly access high-quality data and share their insights without concerns of being “scooped.”

All sought to balance their interests with the societal benefit of participating in N3C and the urgency of addressing the pandemic. The workstream meetings provided a forum for engagement and cooperation, trade-offs, and compromises. Additional input was obtained from the NIH Tribal Health Research Office following Tribal Consultations. The sustained multilateral engagement was crucial to achieving the desired equilibrium in governance.

**2. Step to Success: Establishing a robust yet streamlined data sharing governance that is easy to implement**

To expedite adoption, the Governance Workstream created an agile governance framework. It streamlined its implementation by decoupling the data transfer (DTA) and data use (DUA) agreements, encouraging the use of a single IRB, accepting data in four model formats, and providing scripts to facilitate data extraction and transfer. Separating the DTA and DUA enabled prompt data contribution and recognized that some scientific partners might not be data contributors (though many contributing institutions are both data contributors and accessors).

Institutions needed to obtain IRB authorization for contributing data to the enclave. They could use their local IRB or rely on the John Hopkins University (JHU) IRB as the single IRB of record. The latter option was facilitated using the SMART-IRB Master Reliance Agreement [30]. Adopting a single IRB on a national scale was crucial to harmonizing and accelerating regulatory reviews. Of the more than 230 institutions contributing data, only 3 opted to rely on their own IRB. The JHU IRB granted a waiver of consent for data contribution and authorized the inclusion of children’s data without parental consent and child assent. The local IRBs either agreed with the JHU IRB or determined that participation in N3C was an exempt human subject research.

**3. Step to Success: Robust technical framework that enhances privacy protection**

The N3C is a unique data repository that prioritizes privacy protection to an unprecedented degree by leveraging multiple factors. In 2020, the best cloud vendor was chosen after carefully considering the analytical capabilities and security features required to protect the patient privacy of such a vast population. The software product takes a security-first stance where permission must be obtained rather than have capabilities stripped away. The selected enclave also allows access through popular languages for querying databases, such as R, Python, and SQL. Additionally, the platform can integrate third-party datasets, for example, to enrich social determinants of health data.

N3C also explored novel approaches, including generating synthetic data derivatives via a synthetic data-generating vendor system, developing ways to identify and handle duplicate data records, and optimizing data usability while strictly following privacy-preserving techniques. They engaged experts in deidentification and ETL work to screen for patient data that might have made it through local sites file generation. N3C removed unnecessary barriers to accessing aggregate data but required additional approvals from a local IRB prior to DAC approval to access data containing real dates and full zip codes or using the PPRL-enhanced data elements. Overall, the platform demonstrates a strong interplay between technical and governance layers to ensure the security of EHR data held in trust by the consortium.

**4. Step to Success: Fostering Team Science through behavioral norms, recognition, and reward**

The COVID-19 pandemic brought clinical research and policy questions shared by multiple N3C stakeholders. This overlap in objectives made it highly likely that numerous researchers would pursue similar aims. Researchers self-organized in domain teams to avoid unnecessary redundancies and collaborated to optimize their efforts. This type of large collaborative network was fairly new to biomedical informaticians, clinicians, and researchers before the pandemic, so the Governance Workstream recognized that a positive and collaborative culture would be crucial to the success of this open research team model. The N3C Community Guiding Principles provide these emergent teams with a clear vision of successful team science by outlining behavioral expectations and promoting the values of Partnership, Inclusivity, Transparency, Reciprocity, Accountability, Security, and Mutual Respect.

To ensure appropriate recognition, based on ICMJE recommendations [28], the Publication Committee confirmed that all those responsible for the foundation of the work in any given manuscript were recognized as consortial authors under the author “N3C consortium.” This is facilitated by the software infrastructure of the enclave that allows for objectively and transparently tracking the use of artifacts and attributing credit to contributors. Consortial authors are indexed in PubMed, even though they do not appear individually in the manuscript masthead authors list. While common in environmental science or physics fields, this approach to authorship attribution is less frequent in biomedical research, and some scientific journals have been hesitant to support it, especially for lengthy lists of consortial authors that outpace the journal’s submission mechanisms. N3C also established its own Google Scholar author status with over 195 publications with over 2000 citations [31].

The N3C governance includes additional protections to assess the potential for harm due to publishing analysis output. Concerns about data misuse for political or other ends are addressed through a combination of binding agreements, expected behavioral norms, and oversight mechanisms (i.e., reviews of Data Use Requests, output reviews by the Result Download Committee, and compliance verification by the Publication Committee.

## Lessons learned/ takeaways

The process of developing the N3C Governance ecosystem was imperfect, with significant time and effort required to work through details. We focused on justifying the end result rather than explaining the rationale behind each step along the way. The following key lessons may be useful for others:

1. **Governance is a collective choice**: Governance must balance the interests of stakeholders beyond just data providers, stewards, and users and requires consultations with affected communities and stakeholders to establish the ethical principles and community norms that are the foundation of trust.
2. **Attribution is transitive**: New knowledge builds from prior knowledge. Data scientists’ efforts in data QC, harmonization, preparation for analytics, and interoperability are linked to the quality of analysis outcomes. These efforts should be recognized through proper attribution that leads to funding and career advancement. There must be incentives to participate in every part of shared research and dedicated funding for applying the FAIR guiding principles and for manuscript preparation.
3. **Policies alone aren't sufficient**: The combination of high security, data tiering, clear expectations, and auditing transparency encourages appropriate behavior. All combined, the interactions of policies, robust procedures, and reliable technology facilitate greater cultural adoption of broad access, including access for researchers from other countries invited to collaborate on a DUR led by a US-based investigator and access to aggregate data by citizen scientists.
4. **Aligning responsibility, accountability, and authority is desirable.** Reducing potential incidents is a shared responsibility. However, one must align responsibility, accountability, and authority. For example, NIH is responsible and accountable for the N3C data security (through the N3C data enclave), and the oversight of data use requests. The research community is making decisions on data harmonization. The NIH Result Download Committee does the final approval of all results download requests, while the community-based Publication Committee is responsible for reviewing N3C manuscripts and other research products that contain the NIH-approved results downloads.
5. **Ease of implementation and efficiency support equitable team science**: Communication and coordination are necessary to expedite research and maximize efficiency. For example, the Domain Team structure helps expedite research by limiting the risks of duplication of efforts, but it comes with the responsibility to foster inclusion and the recognition of rights. Similarly, encouraging author attribution supports professional recognition but requires a significant management effort.
6. **Public visibility and inclusion in governance do not undermine the process:** Open governance meetings encouraged dialog and transparency that enhanced rather than impeded governance decision-making.

## Conclusion

A decentralized balanced approach to rule-making can be challenging in aligning expectations but is worthwhile, as demonstrated by the achievement of N3C governance. N3C’s success shows that researchers, institutions, industries, government, and communities can collaborate to establish responsible data sharing and management practices with checks and balances that promote equitable data access, speed up discovery, and maintain public trust. The principles and procedures developed in N3C can be adopted to empower other communities.

## N3C Consortium authors

Amit Saha, Wake Forest Baptist Medical, Winston Salem, NC, USA, Farrukh Koraishy, Stony Brook University, Stony Brook, NY, USA.

## Data partners with release data

The following institutions whose data are released or pending: Available: Advocate Health Care Network – UL1TR002389: The Institute for Translational Medicine (ITM) • Boston University Medical Campus – UL1TR001430: Boston University Clinical and Translational Science Institute • Brown University – U54GM115677: Advance Clinical Translational Research (Advance-CTR) • Carilion Clinic – UL1TR003015: iTHRIV Integrated Translational health Research Institute of Virginia • Charleston Area Medical Center – U54GM104942: West Virginia Clinical and Translational Science Institute (WVCTSI) • Children’s Hospital Colorado – UL1TR002535: Colorado Clinical and Translational Sciences Institute • Columbia University Irving Medical Center – UL1TR001873: Irving Institute for Clinical and Translational Research • Duke University – UL1TR002553: Duke Clinical and Translational Science Institute • George Washington Children’s Research Institute – UL1TR001876: Clinical and Translational Science Institute at Children’s National (CTSA-CN) • George Washington University – UL1TR001876: Clinical and Translational Science Institute at Children’s National (CTSA-CN) • Indiana University School of Medicine – UL1TR002529: Indiana Clinical and Translational Science Institute • Johns Hopkins University – UL1TR003098: Johns Hopkins Institute for Clinical and Translational Research • Loyola Medicine – Loyola University Medical Center • Loyola University Medical Center – UL1TR002389: The Institute for Translational Medicine (ITM) • Maine Medical Center – U54GM115516: Northern New England Clinical & Translational Research (NNE-CTR) Network • Massachusetts General Brigham – UL1TR002541: Harvard Catalyst • Mayo Clinic Rochester – UL1TR002377: Mayo Clinic Center for Clinical and Translational Science (CCaTS) • Medical University of South Carolina – UL1TR001450: South Carolina Clinical & Translational Research Institute (SCTR) • Montefiore Medical Center – UL1TR002556: Institute for Clinical and Translational Research at Einstein and Montefiore • Nemours – U54GM104941: Delaware CTR ACCEL Program • NorthShore University HealthSystem – UL1TR002389: The Institute for Translational Medicine (ITM) • Northwestern University at Chicago – UL1TR001422: Northwestern University Clinical and Translational Science Institute (NUCATS) • OCHIN – INV-018455: Bill and Melinda Gates Foundation grant to Sage Bionetworks • Oregon Health & Science University – UL1TR002369: Oregon Clinical and Translational Research Institute • Penn State Health Milton S. Hershey Medical Center – UL1TR002014: Penn State Clinical and Translational Science Institute • Rush University Medical Center – UL1TR002389: The Institute for Translational Medicine (ITM) • Rutgers, The State University of New Jersey – UL1TR003017: New Jersey Alliance for Clinical and Translational Science • Stony Brook University – U24TR002306 • The Ohio State University – UL1TR002733: Center for Clinical and Translational Science • The State University of New York at Buffalo – UL1TR001412: Clinical and Translational Science Institute • The University of Chicago – UL1TR002389: The Institute for Translational Medicine (ITM) • The University of Iowa – UL1TR002537: Institute for Clinical and Translational Science • The University of Miami Leonard M. Miller School of Medicine – UL1TR002736: University of Miami Clinical and Translational Science Institute • The University of Michigan at Ann Arbor – UL1TR002240: Michigan Institute for Clinical and Health Research • The University of Texas Health Science Center at Houston – UL1TR003167: Center for Clinical and Translational Sciences (CCTS) • The University of Texas Medical Branch at Galveston – UL1TR001439: The Institute for Translational Sciences • The University of Utah – UL1TR002538: Uhealth Center for Clinical and Translational Science • Tufts Medical Center – UL1TR002544: Tufts Clinical and Translational Science Institute • Tulane University – UL1TR003096: Center for Clinical and Translational Science • University Medical Center New Orleans – U54GM104940: Louisiana Clinical and Translational Science (LA CaTS) Center • University of Alabama at Birmingham – UL1TR003096: Center for Clinical and Translational Science • University of Arkansas for Medical Sciences – UL1TR003107: UAMS Translational Research Institute • University of Cincinnati – UL1TR001425: Center for Clinical and Translational Science and Training • University of Colorado Denver, Anschutz Medical Campus – UL1TR002535: Colorado Clinical and Translational Sciences Institute • University of Illinois at Chicago – UL1TR002003: UIC Center for Clinical and Translational Science • University of Kansas Medical Center – UL1TR002366: Frontiers: University of Kansas Clinical and Translational Science Institute • University of Kentucky – UL1TR001998: UK Center for Clinical and Translational Science • University of Massachusetts Medical School Worcester – UL1TR001453: The UMass Center for Clinical and Translational Science (UMCCTS) • University of Minnesota – UL1TR002494: Clinical and Translational Science Institute • University of Mississippi Medical Center – U54GM115428: Mississippi Center for Clinical and Translational Research (CCTR) • University of Nebraska Medical Center – U54GM115458: Great Plains IDeA-Clinical & Translational Research • University of North Carolina at Chapel Hill – UL1TR002489: North Carolina Translational and Clinical Science Institute • University of Oklahoma Health Sciences Center – U54GM104938: Oklahoma Clinical and Translational Science Institute (OCTSI) • University of Rochester – UL1TR002001: UR Clinical & Translational Science Institute • University of Southern California – UL1TR001855: The Southern California Clinical and Translational Science Institute (SC CTSI) • University of Vermont – U54GM115516: Northern New England Clinical & Translational Research (NNE-CTR) Network • University of Virginia – UL1TR003015: iTHRIV Integrated Translational health Research Institute of Virginia • University of Washington – UL1TR002319: Institute of Translational Health Sciences • University of Wisconsin-Madison – UL1TR002373: UW Institute for Clinical and Translational Research • Vanderbilt University Medical Center – UL1TR002243: Vanderbilt Institute for Clinical and Translational Research • Virginia Commonwealth University – UL1TR002649: C. Kenneth and Dianne Wright Center for Clinical and Translational Research • Wake Forest University Health Sciences – UL1TR001420: Wake Forest Clinical and Translational Science Institute • Washington University in St Louis – UL1TR002345: Institute of Clinical and Translational Sciences • Weill Medical College of Cornell University – UL1TR002384: Weill Cornell Medicine Clinical and Translational Science Center • West Virginia University – U54GM104942: West Virginia Clinical and Translational Science Institute (WVCTSI)Submitted: Icahn School of Medicine at Mount Sinai – UL1TR001433: ConduITS Institute for Translational Sciences • The University of Texas Health Science Center at Tyler – UL1TR003167: Center for Clinical and Translational Sciences (CCTS) • University of California, Davis – UL1TR001860: UCDavis Health Clinical and Translational Science Center • University of California, Irvine – UL1TR001414: The UC Irvine Institute for Clinical and Translational Science (ICTS) • University of California, Los Angeles – UL1TR001881: UCLA Clinical Translational Science Institute • University of California, San Diego – UL1TR001442: Altman Clinical and Translational Research Institute • University of California, San Francisco – UL1TR001872: UCSF Clinical and Translational Science InstitutePending: Arkansas Children’s Hospital – UL1TR003107: UAMS Translational Research Institute • Baylor College of Medicine – None (Voluntary) • Children’s Hospital of Philadelphia – UL1TR001878: Institute for Translational Medicine and Therapeutics • Cincinnati Children’s Hospital Medical Center – UL1TR001425: Center for Clinical and Translational Science and Training • Emory University – UL1TR002378: Georgia Clinical and Translational Science Alliance • HonorHealth – None (Voluntary) • Loyola University Chicago – UL1TR002389: The Institute for Translational Medicine (ITM) • Medical College of Wisconsin – UL1TR001436: Clinical and Translational Science Institute of Southeast Wisconsin • MedStar Health Research Institute – UL1TR001409: The Georgetown-Howard Universities Center for Clinical and Translational Science (GHUCCTS) • MetroHealth – None (Voluntary) • Montana State University – U54GM115371: American Indian/Alaska Native CTR • NYU Langone Medical Center – UL1TR001445: Langone Health’s Clinical and Translational Science Institute • Ochsner Medical Center – U54GM104940: Louisiana Clinical and Translational Science (LA CaTS) Center • Regenstrief Institute – UL1TR002529: Indiana Clinical and Translational Science Institute • Sanford Research – None (Voluntary) • Stanford University – UL1TR003142: Spectrum: The Stanford Center for Clinical and Translational Research and Education • The Rockefeller University – UL1TR001866: Center for Clinical and Translational Science • The Scripps Research Institute – UL1TR002550: Scripps Research Translational Institute • University of Florida – UL1TR001427: UF Clinical and Translational Science Institute • University of New Mexico Health Sciences Center – UL1TR001449: University of New Mexico Clinical and Translational Science Center • University of Texas Health Science Center at San Antonio – UL1TR002645: Institute for Integration of Medicine and Science • Yale New Haven Hospital – UL1TR001863: Yale Center for Clinical Investigation.
